# Supplementary material for: Genetic differentiation of a southern Africa tepary bean (Phaseolus acutifolius A Gray) germplasm collection using high-density DArTseq SNP markers
Source: PLoS One. 2023 Dec 14;18(12):e0295773. doi: 10.1371/journal.pone.0295773 (PMC10721083; doi:10.1371/journal.pone.0295773)
Supplement: Attachment — Submitted filename: Comments.pdf [file pone.0295773.s003.pdf]

The manuscript titled "Genetic differentiation of tepary bean (Phaseolus acutifolius) germplasm collection using genotyping-by-sequencing with high-density Single Nucleotide Polymorphism markers" provides an interesting perspective to deduce the genetic groups in tepary bean germplasm collection using GBS. The authors reported a total of 5 genetic groups. These interesting results added to different similar initiatives, will help accelerate breeding programs by offering the status of population structure in the dataset used. In general, the authors have a logical sequence of analysis, in addition to having a high number of SNP molecular markers despite the limited number of genotypes. However, it is strongly recommended to be more descriptive in the bioinformatic and analytic methodology, and be more informative in the figures.. Also, add more references for better context and discussion. The above in order to have more clarity on how the analyzes were carried out, and thus be more effective in the reproducibility of the results in subsequent works. For all the above reasons, I recommend this manuscript for publication in *Plos One* with minor revision. Thus, the authors should address the following recommendations to improve some aspects before the publication.

Below are some comments on each session of the manuscript:

Abstract

Do the authors summarize the main research question and key findings?
R// In general, the authors summarized the main research question and key finding, but I suggest mentioning the method for selecting 5 sub-populations and adding punctuation signs for more clarity in the cluster list.

Introduction

Do the authors identify other literature on the topic and explain how the study relates to this previously published research?
R// In general, the authors have reviewed the current literature, but I suggest adding the most recent works in the topic. For instance, Bornowski et al. in 2023 published a study on genotyping many accessions of tepary beans. On the other hand, I recommend including more references to analytic tools that can be used in this work related to population structure, etc. Additionally, to provide context on how this study relates to previously published research, I suggest including other works on population stratification using GBS in common beans such as Cortés & Blair 2018 or Dartseq as Valdisser et al 2017; or studies involving interspecific panels (Common Bean × Tepary Bean) like those by Cruz et al. in 2023.

Figures and tables

Are the figures and tables clear and readable? (Keep in mind that depending on the

submission system you're working in, you might have to click a link to view the high-resolution versions of the authors' figures).
R// Yes, but: 1. In Figure 3, please list each subplot and change the Eigenvalues of each PC plot to the variance explained of each. PC. 2. In figure 4 organize from largest to smallest according to each percentage of subpopulation and add the accession code at the bottom. In figure 6 add the percentage of variance explained by each component

Is the presentation appropriate for the type of data being presented?
R// Yes, but please add the reference to the R-package used for plotting. For example, did the authors use the CMplot function for Figure 2?

Do the figures and tables support the findings?
R// Yes but it's imperative adjusts the figures and tables.

Methods

What experiments or interventions were used?
R// The authors genotyped 78 accessions of tepary bean by means of the GBS methodology using the first version of P. acutifolius, to obtain 10.527 markers after the SNP Quality Control. Then, the authors carried out a genetic diversity and population structure analysis.

Are the experiments or interventions appropriate for addressing the research question?
R// The experiments and the analytic methodology are appropriate for addressing the research question. However, I suggest providing greater clarity regarding the function used for the ancestry analysis by LEA. Was the algorithm employed for this approach SMNF (e.g., SMNF is an optimization of Structure)? The variant calling process also needs a more detailed explanation. It's unclear how this process was conducted. While they used DArTsoft, a software dedicated to DArTseq, it's worth noting that the use of this tool for GBS is not common. Typically, the main bioinformatic approach for GBS involves tools such as GATK, TASSEL, Stacks, etc.

Are conditions adequate and the right controls in place?
R// Yes, they used a diversity panel of tepary beans that include several races and origins.

Is there enough data to draw a conclusion?
R// Yes, but I advert that the using of this panel for future association analysis would need major number of genotypes accessions.

Do the authors address any possible limitations of the research?
R// Yes

Was data collected and interpreted accurately?

R// Yes

Do the authors follow best practices for reporting?
R// No. I suggest including the company name and country when reporting the use of kits and software.

Does the study conform to ethical guidelines?
R// Yes

Could another researcher reproduce the study with the same methods? In other words, have the authors provided enough information to validate the study?
R// Partially, because the authors have not provided with a link to SNP data or the scripts used in R or other software.

Results, discussion, conclusions

Do the results support the conclusions?
Yes, but I recommend specifying some analysis. In that sense, from data science, the visualization of the first two principal components does not suggest an optimal number of components (PCs) but rather it is a clustering validity analysis that suggests the optimal number of clusters.
