# Supplementary material for: Genetic differentiation of a southern Africa tepary bean (Phaseolus acutifolius A Gray) germplasm collection using high-density DArTseq SNP markers
Source: PLoS One. 2023 Dec 14;18(12):e0295773. doi: 10.1371/journal.pone.0295773 (PMC10721083; doi:10.1371/journal.pone.0295773)
Supplement: Attachment — Submitted filename: II Response to PLoS REVISED.docx [file pone.0295773.s004.docx]

November 11, 2023

Professor Emily Chenette

Editor-in-Chief, PLOS ONE

Dear Prof. Emily Chenette

**Submission of a revised manuscript ID: PONE-D-23-28131**

Thank you very much for the detailed feedback and remarks provided by the reviewers. These have assisted in improving our manuscript ID: **PONE-D-23-28131** entitled "Genetic differentiation of tepary bean (*Phaseolus acutifolius*) germplasm collection using genotyping-by-sequencing with high-density Single Nucleotide Polymorphism markers".

We have carefully made the required revisions using yellow highlighted text in the present submission. Below, please find point-by-point responses and amendments carried out following the reviewers' and editor’s comments:

**With best regards,**

**Saul Eric Mwale**

**Corresponding Author**

Response to PLoS-ONE Comments

Abstract

Reviewer comments: I suggest mentioning the method for selecting 5 sub-populations and adding punctuation signs for more clarity in the cluster list.

Response: Thank you very much for your comments. The method of selecting the 5 sub-populations has been specified in the manuscript and punctuation signs have been added to aid in clarity. The five sub-populations were identified using sparse non-negative matrix factorization (snmf), which computes an entropy criterion (Alexander & Lange, 2011). The entropy criterion helped in choosing the number of ancestral populations that explained the genotypic data. This was further validated using the elbow reference, which involved scree plot of the percentage of variation explained by each component of the PCA. The number of ancestral populations is closely linked to the number of PCA (Byun et al., 2017).

**Reference:**

Alexander, D. H., & Lange, K. (2011). Enhancements to the ADMIXTURE algorithm for individual ancestry estimation. *BMC Bioinformatics*, *12*. https://doi.org/10.1186/1471-2105-12-246

Byun, J., Han, Y., Gorlov, I. P., Busam, J. A., Seldin, M. F., & Amos, C. I. (2017). Ancestry inference using principal component analysis and spatial analysis: A distance-based analysis to account for population substructure. *BMC Genomics*, *18*(1), 1–12. https://doi.org/10.1186/s12864-017-4166-8

Reviewer comments: I suggest adding the most recent works in the topic. For instance, Bornowski et al. in 2023 published a study on genotyping many accessions of tepary beans. On the other hand, I recommend including more references to analytic tools that can be used in this work related to population structure, etc. Additionally, to provide context on how this study relates to previously published research, I suggest including other works on population stratification using GBS in common beans such as Cortés & Blair 2018 or Dartseq as Valdisser et al 2017; or studies involving interspecific panels (Common Bean × Tepary Bean) like those by Cruz et al. in 2023.

Response: The suggested articles have been added, and our study findings have been contextualized in line with published literature. We have included DArTseq, a more specific GBS platform used in the current study, for clarity.

Reviewer comments: In Figure 3, please list each subplot and change the Eigenvalues of each PC plot to the variance explained of each. PC. 2. In figure 4 organize from largest to smallest according to each percentage of subpopulation and add the accession code at the bottom. In figure 6 add the percentage of variance explained by each component.

Response: Figure 3: We have plotted a PC scree plot of PCs against the variance, explained as suggested. However, we could not change the PC into subpopulations because, in this method, the number of PCs are the subpopulations. By looking at the scree plot, the PCs can reliably inform us of the optimum number of K.

Response: Figure 4: As suggested, we have added accession codes at the bottom of the plot. With your advice, we have refined the admixture plot, and the groups are better visible.

Response: Figure 6: We have added percentage variation on each coordinate.

Reviewer comment: R//Yes, but please add the reference to the R-package used for plotting. For example, did the authors use the CMplot function for Figure 2?

Response: We have cited all referenced R packages used, including CMplot.

Reviewer comment: *Are the experiments or interventions appropriate for addressing the research question?*

R// The experiments and the analytic methodology are appropriate for addressing the research question. However, I suggest providing greater clarity regarding the function used for the ancestry analysis by LEA. Was the algorithm employed for this approach SMNF (e.g., SMNF is an optimization of Structure)? The variant calling process also needs a more detailed explanation. It's unclear how this process was conducted. While they used DArTsoft, a software dedicated to DArTseq, it's worth noting that the use of this tool for GBS is not common. Typically, the main bioinformatic approach for GBS involves tools such as GATK, TASSEL, Stacks, etc.

Response: Thank you very much for your comment. We have provided more information on the function snmf as used in the LEA package to estimate the co-ancestry coefficient of the population. The current GBS was done using the DArTseq platform, which is reliably analyzed using DArTsoft 14. DArTsoft is an automated genotypic data analysis program that was developed to analyze sequence data generated from DArTseq (Gelaw et al., 2023; Adu et al., 2021; Alam et al., 2018).

Reviewer comment: Is there enough data to draw a conclusion? R// Yes, but I advert that the using of this panel for future association analysis would need major number of genotypes accessions.

Response: Thank you for the comment. We will consider including several accessions in future association analysis.

Reviewer comment: Do the authors follow best practices for reporting? R// No. I suggest including the company name and country when reporting the use of kits and software.

Response: We have included the company for the TANBEAD Plant Extraction Kit (Diagnocine, Hackensack, NJ, USA) and a QIAquick PCR purification kit (QIAGEN GmbH, Hilden, Germany).

Reviewer comments: Could another researcher reproduce the study with the same methods? In other words, have the authors provided enough information to validate the study? R// Partially, because the authors have not provided with a link to SNP data or the scripts used in R or other software

Response: Supporting information on genotypic data has been provided.

Reviewer comments: Do the results support the conclusions? Yes, but I recommend specifying some analysis. In that sense, from data science, the visualization of the first two principal components does not suggest an optimal number of components (PCs) but rather it is a clustering validity analysis that suggests the optimal number of clusters.

Response: Thank you for the comment. The cross-entropy criteria was used to identify the optimum number of K populations and the identified subpopulations were validated by principal component analysis.
